# Impact of Phenolic Acid Derivatives on the Oxidative Stability of β-Lactoglobulin-Stabilized Emulsions

**DOI:** 10.3390/antiox12010182

**Published:** 2023-01-12

**Authors:** Alina Bock, Helena Kieserling, Ulrike Steinhäuser, Sascha Rohn

**Affiliations:** 1 Department of Food Chemistry and Food Analysis, Institute of Food Technology and Food Chemistry, Technische Universität Berlin, Gustav-Meyer-Allee 25, 13355 Berlin, Germany; 2 Department of Food Technology and Food Analysis, Berliner Hochschule für Technik, Luxemburger Straße 10, 13353 Berlin, Germany

**Keywords:** phenolic acid derivatives, β-lactoglobulin, oil-water-emulsions, pro-oxidant, transition metal complexation, iron reducing power, radical scavenging, oxidation products

## Abstract

Proteins, such as β-lactoglobulin (β-Lg), are often used to stabilize oil–water-emulsions. By using an additional implementation of phenolic compounds (PC) that might interact with the proteins, the oxidative stability can be further improved. Whether PC have a certain pro-oxidant effect on oxidation processes, while interacting non-covalently (pH-6) or covalently (pH.9) with the interfacial protein-film, is not known. This study aimed to characterize the impact of phenolic acid derivatives (PCDs) on the antioxidant efficacy of the interfacial β-Lg-film, depending on their structural properties and pH-value. Electron paramagnetic resonance (EPR) analyses were performed to assess the radical scavenging in the aqueous and oil phases of the emulsion, and the complexation of transition metals: these are well known to act as pro-oxidants. Finally, in a model linseed oil emulsion, lipid oxidation products were analyzed over storage time in order to characterize the antioxidant efficacy of the interfacial protein-film. The results showed that, at pH.6, PCDs can scavenge hydrophilic radicals and partially scavenge hydrophobic radicals, as well as reduce transition metals. As expected, transition metals are complexed to only a slight degree, leading to an increased lipid oxidation through non-complexed reduced transition metals. At pH.9, there is a strong complexation between PCDs and the transition metals and, therefore, a decreased ability to reduce the transition metals; these do not promote lipid oxidation in the emulsion anymore.

## 1. Introduction

Most processed foods are emulsions. However, emulsions need to be regarded as both physically and chemically labile systems. Physically, emulsions tend to coalesce, to flocculate, and finally, to cream, resulting in phase separation [1]. Chemically, emulsified oil droplets have a large surface area; this favors lipid oxidation by oxygen, and being triggered through other pro-oxidant compounds that are able to diffuse through the aqueous phase onto the interface and potentially into the oil [2]. To stabilize emulsions, amphiphilic compounds, such as (small molecule) emulsifiers or proteins, are used. Proteins can unfold and align themselves with an amphiphilic conformation at the interface with their side chains by changing their secondary and tertiary structure [3]. Due to intermolecular protein interactions, a protein film forms at the interface, stabilizing the oil–water interface, and prevents physical instability [4]. Typical proteins used as emulsifiers are whey proteins or a variation of plant proteins, with legume proteins with soy being the most prominent representative [2].

Emulsifiers and proteins can have antioxidant properties and decrease lipid oxidation, and there is also the possibility of adding other antioxidants in order to reach a higher antioxidant efficacy in the interface to stabilize the emulsion. High antioxidant efficacy can be achieved when present free radicals can be scavenged and the chain reaction in the course of lipid oxidation can be slowed down or inhibited. Typical antioxidants, naturally present in foods, include phenolic compounds such as flavonoids or phenolic acids. They are resonance stabilized by extended π-electron systems, enabling them to split off protons, scavenge radicals, and convert themselves into less reactive radicals [5,6,7]. The property of self-oxidation and electron donation can also be disadvantageous for oxidative emulsion stability, as transition metals present in food can be reduced to pro-oxidant compounds within a redox cycle mode of action [8,9]. However, some studies showed that accelerated lipid peroxidation is due to the pro-oxidant effect of phenolic compounds [10,11,12]. The initial electron transfer oxidation of phenolic compounds, through the reduction of transition metals, generates the corresponding semiquinone radical of the phenolic compound. This semiquinone radical proceeds through a second electron transfer reaction with O_2_ to form an ortho-quinone and a superoxide anion (O_2_^•−^). Then, O_2_^•−^ reacts with reduced transition metals to form hydrogen peroxide (H_2_O_2_). The Fenton reaction can occur as H_2_O_2_ reacts to form a hydroxyl radical (OH^•^). Reactive oxygen species (ROS), such as OH^•^ and O_2_^•−^, are formed and promote lipid oxidation [11,13,14]. Lipid oxidation is initially manifested in the formation of primary oxidation products such as conjugated dienes and hydroperoxides. Then, these react to form secondary oxidation products, such as alkanes, esters, alcohols, ketones, or aldehydes [15]. The secondary oxidation products continue to be chemically reactive compounds that can further react to form tertiary oxidation products. While primary oxidation products are colorless and tasteless, secondary and tertiary oxidation products can lead to off-flavors and, in some cases, be harmful to health [16,17]. The formation of tertiary oxidation products is a sign of an advanced oxidative deterioration [18].

The ability of phenolic compounds to reduce transition metals and, therefore, have a pro-oxidant effect, is contrasted by their ability to complex transition metals. Khokhar et al. showed that phenolic compounds with o-dihydroxyl structures can complex Fe(III) [19]. In this regard, Hider et al. showed that the corresponding catechol structures could bind transition metals in a ratio of 1:1 to 1:3 (transition metal: catechol structure) [20]. This makes it difficult to assess whether phenolic compounds are pro-oxidant through their transition metal-reducing capacity or antioxidant through their transition metal complexation in an emulsion.

While transition metals, due to their charge, are mainly located in the aqueous phase of the emulsion or at the interfacial film, if there is no electrostatic repulsion to the interfacial film, phenolic compounds can partition themselves between the oil, interface and water phase, depending on their structural properties. The structural properties of phenolic compounds mainly result from the molecule size, the polarity, which in turn is affected by polar substituents, such as hydroxyl or glycosyl groups, as well as the pH, depending on charge [21]. Thus, the partitioning behavior of the phenolic compounds further has an impact on their anti- and pro-oxidant properties, because charged transition metals in the aqueous phase of the system would have to be reduced in the aqueous phase in order to have a pro-oxidant effect [8]. In addition, radicals already present in an emulsion can only be scavenged by phenolic compounds, provided that they are located in the same phase of the emulsion.

Another factor that may affect the anti- and pro-oxidant activity of phenolic compounds in the emulsion are the interactions between the phenolic compounds and the interfacial protein(s). Depending on the conditions, such as the pH value, temperature, ionic strength, and the presence of chemical reagents or food components, these interactions can be non-covalent or covalent interactions [21]. The effect of non-covalent and covalent interactions between phenolic compounds and proteins on their antioxidant properties has been mainly studied in aqueous systems [22,23,24].

In some studies, phenolic compounds and proteins were shown to interact primarily in the aqueous phase and were, then, used for emulsification [25,26]. However, these results presented a mixed picture of the antioxidant effect of the interactions. For example, some studies showed that the oxidative stability of the emulsion was improved [27,28,29], but in others it decreased the oxidative stability through non-covalent and covalent interactions between the phenolic compounds and the proteins [10,12,21,29]. Little attention has been paid to studies in which the interfacial protein film (having an amphiphilic alignment of the protein side chains in the oil and the water phase) interacts directly (on-site at the interface) with the phenolic compounds and how these interactions affect the oxidative stability of the emulsion.

Consequently, this study aimed to characterize the impact of phenolic acid derivatives (PCDs), depending on their chemical–structural properties and interaction conditions (pH value), on the antioxidant efficacy of the interfacial protein film and the resulting oxidative emulsion stability.

To investigate this issue, four hypotheses were conceived: (1) PCDs with a high polarity and thus, high-water solubility, exist in a dissolved state in the aqueous phase of the emulsion or interact with the non-polar side of the interfacial film. From there, PCDs can scavenge radicals from the aqueous phase. (2) Secondly, it is assumed that low molecular weight PCDs, with a non-polar molecular character and thus, a low water solubility, partially diffuse from the aqueous phase of the emulsion through the interfacial film. There, they can interact with the hydrophobic side of the interfacial film or are (partly) dissolved in the lipid phase. From there, PCDs can scavenge lipid radicals present in the oil. (3) The third hypothesis assumes that, depending on the pH, PCDs can maintain the redox cycle in food by reducing Fe(III) to pro-oxidant Fe(II). At pH 6, PCDs interact predominantly non-covalently with the interfacial protein film. The molecular structure of PCDs are, therefore, not modified through covalent bonds; this, in turn, makes it easier for them to split off electrons and reduce Fe(III). Fe(II), in turn, promotes the further reaction of hydroperoxides to secondary oxidation products within an emulsion. (4) It is assumed that at pH 9, PCDs exist in a polymerized state or covalently bound to the interfacial film, which reduces the ability of electron splitting and thus, the Fe reduction power. In addition, the hydroxyl groups of the PCD deprotonate with an increasing pH, which can complex transition metals as a result. Complexed transition metals are, then, no longer available to the redox cycle and lose their pro-oxidant effect. The further reaction of hydroperoxides to secondary oxidation products within an emulsion is therefore not supported. Therefore, the radical scavenging of PCDs in an emulsion was studied by electron paramagnetic resonance (EPR) spectroscopy at pH 6 and 9 for hydrophilic und hydrophobic radicals. The ability of the system to complex transition metals was also characterized by EPR. In a next step, the oxidative stability of a Fe-containing linseed oil emulsion was checked over a storage time of 21 days by detecting primary, secondary, and tertiary oxidation products via photometric, potentiometric and gas chromatographic methods.

## 2. Materials and Methods

### 2.1. Phenolic Acid Derivatives—Structural Characteristics

**Figure 1:**
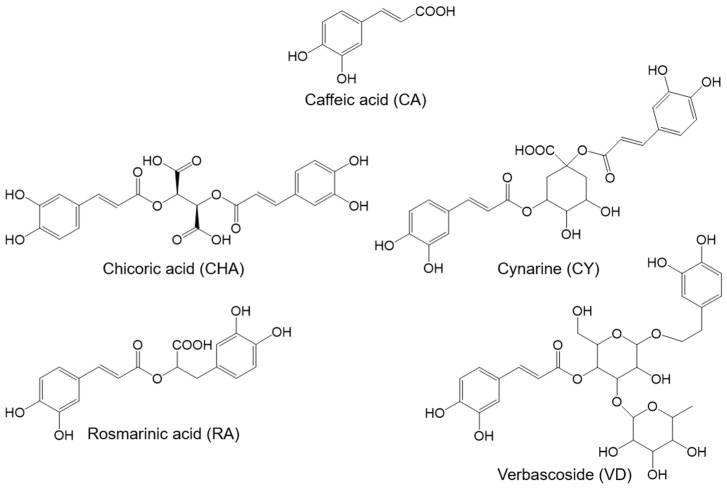
Structures of the added phenolic acid derivatives.

All chosen phenolic acid derivatives (PCDs) were based on caffeic acid (CA) (Figure 1). Chicoric acid (CHA) and cynarine (CY) contain two caffeic acid units, while rosmarinic acid (RS) and verbascoside (VD) contain one caffeic acid unit and a substituted catechol-group. CY and VD are esterified with polar substituents, such as quinic acid and rhamnose/glucose. All PCDs were purchased from Carl Roth GmbH & Co. KG (>98%; Karlsruhe, Germany).

### 2.2. Phenolic Acid Derivative Solutions

PCD solutions were prepared in distilled water with concentrations of 0.895 mM. For dissolving the PCDs, an ultrasonic bath was used for 15 min. To prevent polymerization reactions in the PCD solutions, these were prepared freshly, and pH values were set to 6.0 or 9.0 directly before adding them to the emulsion at room temperature. The pH-value was set with 0.1 M of HCl and 0.1 M of NaOH (>99.9%; analytical grade, Carl Roth GmbH & Co. KG, Karlsruhe, Germany).

### 2.3. β-Lactoglobulin Solutions

Native β-lactoglobulin (β-Lg) was purified from a whey protein isolate (Davisco Foods International Inc.; Le Sueur, MN, USA) to 99%, as described by Keppler et al. [30]. An aqueous β-Lg-solution was prepared with distilled water to obtain a β-Lg concentration of 0.1 weight percent (wt.%) in the emulsion. For this purpose, the dissolved β-Lg was stirred at room temperature for 1 h and the pH value was set to 6.0 or 9.0, with 0.1 M of HCl and 0.1M of NaOH (>99.9%; analytical grade, Carl Roth GmbH & Co. KG, Karlsruhe, Germany).

### 2.4. Oil Purification

MCT oil (medium-chain-triacylglycerols, >99.9%; ENDIMA GmbH, Illingen, Germany) was used for EPR-measurements. Linseed oil (cold pressed organic linseed oil; Biovenue!, Montpellier, France), which is rich in unsaturated fatty acids and rich in transition metals, such as iron (0.34 mg/100 g), zinc (0.31 mg/100 g), and copper (0.06 mg/100 g), was used [31]. It was used in this study to allow the competitive effects of the antioxidant and the pro-oxidant effects of the PCDs to be investigated in a linseed oil model emulsion. To remove oil-accompanying substances, the oils were purified with Florisil^®^ (100%; MgO × 3.6 SiO2 × 1.53 OH, Carl Roth GmbH & Co. KG, Karlsruhe, Germany) in a ratio of 3:1 (oil: Florisil^®^). The oil was agitated with a magnetic stirrer for at least 2 h at room temperature and, then, centrifuged at 10,000× *g* for 45 min.

### 2.5. EPR-Characterization of Copper Complexation in the Emulsion

To characterize the copper complexation properties of the emulsion, emulsions were prepared in 200 µL micro mixtures containing 0.1% β-Lg and 10% MCT oil in their final concentration. Copper sulfate pentahydrate (>99%; Merck KGaA, Darmstadt, Germany) was added to the samples so that the final sample concentration of Cu(II) was 2.5 mM. The actual emulsification process was carried out in an ultrasonic bath for three minutes. Then, 20 min after emulsification, a PCD was added, and the pH was adjusted to 6 or 9. The PCD concentration was 5 mM in the final sample. EPR measurements were performed with an internal laboratory protocol on a MiniScope MS200 (magnettech GmbH, Berlin, Germany) after an incubation period of 1 h. The following parameters were used for this: B0-field: 330 mT, range: 80 mT, sweep time 30 s, number of passes: 15, modulation: 0.2 mT, microwave (MW) attenuation: 10.0 db and gain 300. The sample collection for the measurement was carried out in disposable capillary pipettes (ringcaps 50 µL, Hirschmann Laborgeräte GmbH, Eberstadt, Germany).

### 2.6. Radical Degradation from the Aqueous Phase of the Emulsion

To characterize the radical degradation kinetic from the aqueous phase of the emulsion, emulsions were prepared in an ultrasonic bath for three minutes containing 0.1% β-Lg and 10% MCT oil in their final concentration. Then, 20 min after emulsification, a PCD (0.895 mM) was added in a molar ratio (β-Lg:PCD) 1:10, the pH was adjusted to 6 or 9, and the emulsion was incubated for one hour. After incubation time, 20 µL of the free hydrophilic radical 4-hydroxy-TEMPO (tempol, 0.054 mM dissolved in distilled water; 97%; Merck KGaA, Darmstadt, Germany) was added to the 180 µL emulsion and dispersed using a vortex mixer. Measurements started immediately afterwards.

The EPR kinetic measurement was performed for 30 min, with a measurement every 60 s, using the following parameters: B0-field: 450 mT, range: 10 mT, sweep time: 30 s, number of passes: 1, modulation: 0.2 mT, MW attenuation: 10.0 db and gain 70.

### 2.7. Radical Degradation from the Lipid Phase of the Emulsion

To characterize the radical degradation kinetics of the lipid phase of the emulsion, the PCD (0.895 mM) and β-Lg (0.2 wt.%) solutions were mixed, the pH-values were set to 6.0 or 9.0, and the samples were incubated for 1 h. After incubation time, 80 µL of the hydrophobic free radical 16-doxyl stearic acid (0.054 mM dissolved in MCT-oil; Merck KGaA, Darmstadt, Germany) was mixed with a 180 µL PCD–β-Lg mixture using a vortexer. After vortexing, emulsions were prepared in an ultrasonic bath for 1 min and measured directly after preparation.

The EPR kinetic measurement was performed for 30 min, with a measurement every 60 s, using the following parameters: B0-field: 350 mT, Range: 50 mT, Sweep time: 30 s, Number of passes: 1, Modulation: 0.2 mT, MW attenuation: 10.0 db and Gain 70. This method was a self-established internal laboratory protocol.

### 2.8. Iron Reducing Power

The reducing power of the aqueous PCD–β-Lg mixtures was studied with a modified method described by Oyaizu (1986) [32]. First, the β-Lg solution (0.1 wt.%) was incubated with the PCD in a molar ration of 1:3 (β-Lg:PCD) at pH 6.0 or pH 9.0 for one hour while stirring the mixture. A volume of 2 mL of the aqueous PCD–β-Lg mixtures, or an ascorbic acid (99.7%; Merck KGaA, Darmstadt, Germany) calibration standard (10–60 mg/L), were mixed with 2 mL of the phosphate buffer (0.2 M, pH 6.6; Merck KGaA, Darmstadt, Germany) and 2 mL of potassium hexacyanoferrate (1%; Merck KGaA, Darmstadt, Germany). The samples were incubated in a water bath at 50 °C for 20 min. Then, 2 mL of trichloroacetic acid (10%; Carl Roth GmbH & Co. KG, Karlsruhe, Germany) were added and the samples were centrifuged at 1,500× *g* for 10 min. For the photometric measurement, 1.3 mL of the supernatant was mixed with 0.26 mL of iron (III) chloride (0.1 %; neoLab^®^ Migge GmbH, Heidelberg, Germany) and 1.3 mL of distilled water. The photometric measurement was, then, performed exactly 10 min after mixing the reagents in the cuvette at 700 nm [33].

### 2.9. Emulsification and Incubation of the Linseed Oil Emulsion

For the storage tests, an emulsion was prepared containing 10% linseed oil as the final concentration and stabilized with 0.1 wt.% β-Lg. An oil–water stock emulsion was prepared for this purpose. A premix was created by mixing 140 g of 0.1 wt.% β-Lg solution and 70 g of purified linseed oil using a rotor–stator system (Ultra-Turrax^®^ T25 basic, IKA -Werke GmbH & CO. KG, Staufen, Germany) at 13,500 rpm for 60 s. The premix emulsion was homogenized in a high-pressure homogenizer (APV 1000, APV Systems Denmark) at 300 bar with 2 passes. The oil droplet size was measured by static light scattering (LS 13 320 Particle Size Analyzer, Beckman Coulter GmbH, Krefeld, Germany). After emulsification, sodium azide (0.02% in water) was added to prevent microbial growth. The stock emulsion was halved and set to pH 6.0 and 9.0, and split in 8 g samples. Then, 20 min after emulsification, PCD solutions (adjusted to pH 6.0 or 9.0) were added to obtain a β-Lg:PCD molar ratios of 1:10; this was stirred with a glass rod. The storage of the samples took place at room temperature and with sunlight for 21 d.

### 2.10. Determination of Hydroperoxide Values in the Linseed Oil Emulsion

To extract the oil out of the emulsion, 0.7 g of emulsion was mixed with 2.5 mL of distilled water and 7 mL of an isopropanol/ isooctane (1:1; *v*:*v*) mixture (>99.9%; Carl Roth GmbH & Co. KG, Karlsruhe, Germany). This mixture was vortexed for 30 s to solve the oil in the organic solvent. For a better water–solvent separation, the samples were centrifuged at 7,500× *g* for 5 min. An aliquot of 2.5 mL of the organic solvent supernatant were evaporated under a nitrogen flow at 40 °C. The leftover oil was weighed, resolved in 30 mL of an acetic acid/chloroform (3:2; *v*:*v*) mixture (100%/ >98%; VWR International GmbH, Darmstadt, Germany), and transferred to an Erlenmeyer flask. A volume of 0.5 mL of saturated potassium iodide (>99.5%; Th. Geyer GmbH, Renningen, Germany) solution was added to the Erlenmeyer flask. The sample was stirred for 60 s. Then, 30 mL of distilled water was added. This was followed by an automated potentiometric titration (TitroLine^®^ 7000, Pt 61 electrode, Xylem Analytics Germany Sales GmbH & Co. KG, Weilheim, Germany) with sodium thiosulphate (0.001 N; Merck KGaA, Darmstadt, Germany) [18].

### 2.11. Determination of Conjugated Dienes in the Linseed Oil Emulsion

Conjugated dienes were determined following a modified method, described by Mei et al. (1998). For this purpose, 100 µL of isopropanol/isooctane oil extract was taken from the analysis in Section 2.10 and mixed with 2.7 mL of solvent (methanol/butanol; 2:1; *v*:*v*; >99.9%; Carl Roth GmbH & Co. KG, Karlsruhe, Germany). This mixture was transferred to a fused silica cuvette and the absorbance was measured at 233 nm on a UV-Vis spectrophotometer (photoLab6600 UV-Vis, Xylem Analytics Germany Sales GmbH & Co. KG, Weilheim, Germany). The increase in the absorbance at 233 nm is characteristic of the conjugated diene formation [34].

### 2.12. GC-Analysis of Secondary Oxidation Products in the Linseed Oil Emulsion

Secondary lipid oxidation products were quantified after a 21 d storage time by solid phase microextraction (SPME) and gas chromatography (GC), coupled to mass spectrometry (MS). 2-Heptanone (<98%; Merck KGaA, Darmstadt, Germany) was added as an internal standard. The SPME-fibers were put in 20 mL-headspace vials and filled with 8 g of sample (emulsion from Section 2.9). The vials were sealed with a polytetrafluoroethylene (PTFE)-septa. The extraction/adsorption was performed at 60 °C for 10 min. The desorption of the analytes from the SPME fiber takes place at 250 °C for 120 s. The system featured a splitless injection and flow of 2 mL/min with helium. The stationary phase used a medium polar Zebron ZB-1701 capillary column (60 m × 0.32 mm × 0.25 µm) (Phenomenex Inc., Torrance, CA, United States). The temperature program started with a temperature of 40 °C for 5 min, and was, then, raised by 2 °C/min, up to 60 °C. After a 2 min holding time, the temperature was raised at a rate of 10 °C/min, up to 120 °C. In the last step, the temperature was increased at a rate of 40 °C/min, up to 260 °C, and held for 10 min. For detection, electron ionization mass spectrometry was performed at 70 eV [35].

### 2.13. Statistic Analysis

All measurements were performed in triplicate, except for the qualitative EPR measurements. Following a Shapiro–Wilk test for normal distribution, a one way ANOVA test with a Tukey HSD post hoc (*p* < 0.05) test was conducted with xlstat (Addinsoft SAS, Bordeaux, France) to identify significant differences between the samples. The order of the statistical differences used in this analysis is *a* > *b* > *c*. The homogeneity of variances was assumed for this purpose.

## 3. Results and Discussion

The first step was to investigate, by EPR, whether PCDs can directly scavenge radicals at pH 6 and 9 from the oil or water phase of a β-lactoglobulin (β-Lg)-stabilized emulsion. β-Lg was chosen as a model protein, because its interfacial properties have been well studied and, as a whey protein, it is frequently applied in foods [3,36,37,38]. In the second step, EPR was used to characterize the complexation of transition metals by the interfacial β-Lg film containing PCDs. For this purpose, the extent to which PCD–β-Lg mixtures can reduce transition metals was investigated. In the third step, the impact of the different structured PCDs and the pH value on the formation of primary, secondary and tertiary oxidation products was determined in a ferrous linseed oil emulsion, over a storage period of 21 d.

### 3.1. Radical Scavening of Hydrophilic and Hydrophobic Radicals

The ability of interfacial films to scavenge the free radicals that may start the chain reactions during the lipid oxidation process has been studied in two different ways with the use of a hydrophilic and a hydrophobic radical. Both free radicals in the aqueous phase of the emulsion and in the lipid phase can maintain the chain reaction of lipid oxidation. To imitate a comparable system, a hydrophilic and a hydrophobic free radical were used to investigate in which phase of the emulsion radicals can be scavenged by the interfacial film via EPR.

**Figure 2:**
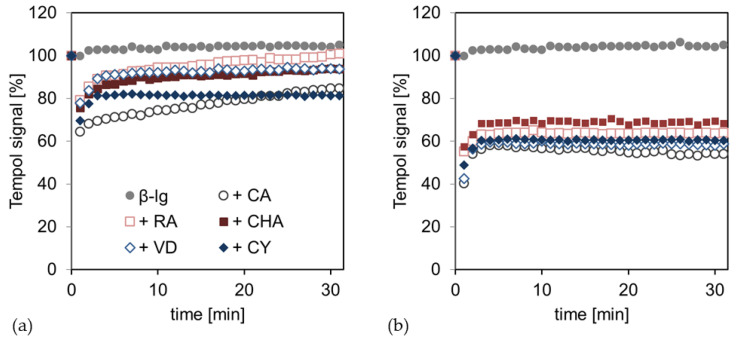
Tempol degradation kinetic from the aqueous phase (**a**) of the emulsion at pH 6 and (**b**) at pH 9; here, the β-Lg emulsions are compared without (β-lg) and with PCD (+ PCD) addition. The starting point at t = 0 min represents the output signal of the pure radical. From t = 1 min onwards, the radical degradation or the radical regeneration is shown by an increase or decrease in the original radical signal. The values shown are an individual exemplary series of measurements.

Figure 2 shows the time-dependent radical scavenging of the hydrophilic free radical tempol by the PCD-crosslinked interfacial films at pH 6 and 9. The emulsions without PCD did not show any radical scavenging, whereas the addition of PCD led to a significant initial decrease in the radical signal at both pH values. At pH 6, the PCD addition could initially decrease the tempol signal by about 30%; this was, therefore, approximately 70% of the initial tempol signal. In the following time course, an asymptotic increase in the tempol signal could be observed for samples containing PCD. For the hydrophobic PCD, CA and CY, this increase was less pronounced; there was approximately 10% of the initial tempol signal in 30 min, while the hydrophilic PCD, RA, CHA, and VD showed an increase of 20% or more in 30 min. These were, therefore, almost at the initial level.

At pH 9, the tempol signal was initially reduced by approximately 50–60% with the addition of PCD, and was thus approximately 40–50%. Comparable to pH 6, an asymptotic increase in the tempol signal of about 20% in 30 min was observed over the time course, but no significant differences between the PCDs were observed.

The pH value, therefore, appears to have a significant impact on the extent to which the PCDs can scavenge free radicals. The ability of the PCDs to deprotonate various phenolic hydroxyl groups increases with rising pH and, therefore, the possibility of radical scavenging, as previously shown for catechins [39] and anthocyanins [40].

The observed increase in the radical signal, following tempol degradation at both pH levels, suggests that tempol radicals, previously scavenged by PCDs, are regenerated under ambient conditions in the emulsion. The scavenged tempol-H turns into the free radical tempol again. This is possible because the addition of radicals in the emulsion methodically deposited oxygen in through vortexing. As Monti et al. have shown, tempol-H is able to be regenerated into the free radical tempol by oxygenation in a very short time [41].

At pH 6, different properties of the PCDs, with regard to the radical scavenging activity, can be identified, depending on their chemical structure. It is striking that PCDs with the highest hydrophobicity and thus, a low water solubility, most strongly prevent tempol-H from regenerating; this is compared to hydrophilic PCDs with a high water solubility. This observation is, in principle, in line with previously described research results, stating that moderately hydrophobic antioxidants are particularly effective in emulsions. This observation is mainly explained by the accumulation of moderately hydrophobic antioxidants at the interface [42,43]. This explanation does not apply to the present study, as the hydrophobicity of the PCDs did not affect radical scavenging per se, but radical regeneration.

At pH 9, no differences in radical scavenging and regeneration, as well as in the deviating hydrophobicity of the PCDs, can be observed due to the chemical–structural differences. This observation is probably due to the polymerization reactions of the PCDs at pH 9; this is because the polymers have more similar chemical properties than the monomers, where individual substituents are more important. This phenomenon has already been noticed in a previous study on physical emulsion stability [44].

**Figure 3:**
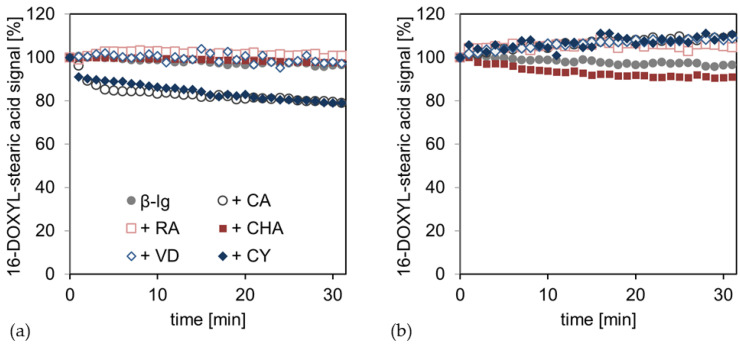
16-DOXYL-stearic acid degradation kinetic from the lipid phase (**a**) of the emulsion at pH 6 and (**b**) at pH 9; Here, the β-Lg emulsions are compared without (β-lg) and with PCD (+PCD) addition. The starting point at t = 0 min represents the output signal of the pure radical. From t = 1 min onwards, the radical degradation or radical regeneration is shown by an increase or decrease in the original radical signal.

Figure 3 shows the time-dependent radical scavenging of the hydrophobic free radical 16-DOXYL-stearic acid by the PCD-crosslinked interfacial films at pH 6 and 9. At pH 6, the pure β-Lg interfacial film, as well as those crosslinked with hydrophilic PCD (RA, CHA, and VD), showed no scavenging of hydrophobic radicals. However, the addition of the hydrophobic poorly water-soluble PCDs (CA and CY) resulted in nearly identical radical scavenging kinetics, where the radical signal was reduced by approximately 20% in 30 min. In contrast, the samples at pH 9 showed no significant tendencies to scavenge the hydrophobic radical from the lipid phase.

At pH 6, the radical scavenging of CA and CY from the lipid phase can be explained by the molecular properties, such as the hydrophobicity, and their following reaction conditions. In previous studies, it has been shown that CA and CY accumulate more profoundly in the lipid phase of an MCT–oil–water system. The other compounds studied (RA, CHA, and VD) were almost completely present in the aqueous phase [45]. In addition, PCDs are protonated and, thus, have a low net charge at pH 6; this allows CA and CY to transition to the lipid phase. On the other hand, the PCDs are negatively charged at pH 9, which, in turn, shifts the partitioning of the PCDs in the direction of the aqueous phase due to an increasing polarity through the charge [46]. As hypothesized, at pH 6, CA and CY may, therefore, be present freely in the lipid phase or interact with the interfacial film coming from the lipid phase, where they can scavenge hydrophobic lipid radicals. Due to the anionic charge of PCDs at pH 9, PCDs attach to the interfacial film, starting from the aqueous phase or are free in the aqueous phase, so that they are sterically hindered and separated from the hydrophobic lipid radical and cannot scavenge it.

In summary, PCDs experience a higher scavenging of radicals in the aqueous phase of the emulsion at pH 9 than at pH 6, because of the hydroxyl groups being deprotonated and the follow-up reaction releasing protons with free radicals. However, at pH 9, electrostatic repulsion, polymerization reactions of the PCDs, and covalent binding to the interfacial protein film can occur. Consequently, the PCDs are hindered from scavenging hydrophobic radicals from the lipid phase, because they are sterically prevented from penetrating the interfacial film. On the other hand, PCDs have a low net charge at pH 6 and are (small) monomers, so that they can penetrate the interfacial film and saturate radicals in the lipid phase, depending on their polarity.

### 3.2. Complexation of Transition Metals

The complexation of transition metals by β-Lg-PCD mixtures was investigated with an EPR measurement at pH 6 and 9, in order to assess the impact of structural effects and pH on the formation of an interfacial complex between the transition metal Cu(II) and the interfacial β-Lg-PCD mixture.

**Figure 4:**
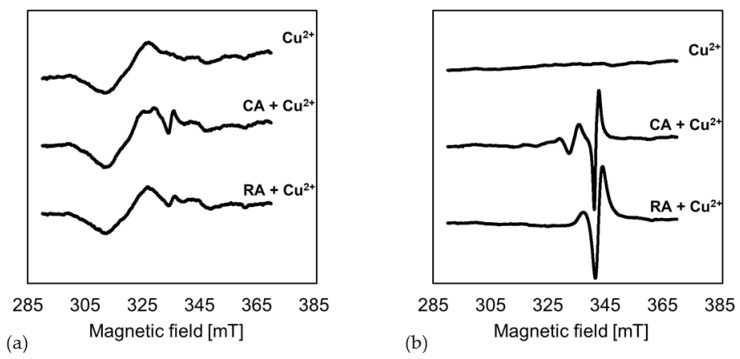
The EPR spectra show the change in the Cu(II) signal in the presence of the β-Lg interfacial film of an emulsion without PCD addition (top) and with the addition of CA (middle) and RA (bottom) at (**a**) pH 6 and (**b**) pH 9. The spectra shown are an individual exemplary series of measurements.

Figure 4 shows the EPR spectra of the interfacial films without PCDs, and with CA and RA, in the presence of Cu(II) at pH 6 and 9. The basic Cu(II) signal presents itself as a wave in the range of 320 mT at pH 6. In the presence of CA and RA, a change in the basic signal in the range of 335 mT was observed, suggesting a partial complexation of Cu(II) by PCDs. At pH 9, the Cu(II) signal was no longer measurable due to the pH value. At pH 9, the dissolved Cu(II) was precipitated as Cu(OH)_2_, which is not EPR active. However, in the presence of CA and RA, the Cu(II) was complexed and thus held in solution. This was shown by a copper complex signal at around 340 mT.

Goodman et al. (2012) were able to demonstrate a pH-dependent complex formation with the formation of different complexes for tea polyphenols and Cu(II). They observed a slight weakening of the Cu(II) signal at pH 6 for ellagic acid and Cu(II); this shows a clear shift in the EPR spectrum with an increase in the pH to 10, due to the formation of a complex [47]. Pirker et al. (2012) described that, at weakly acidic reaction conditions, silent EPR species form, showing almost no change in the spectrum; these are, therefore, difficult to characterize [48]. Ferreira Severino et al. (2011) suggested that such silent EPR species involve the formation of di- or polymeric complexes in which the Cu(II) is coordinated via carboxyl groups [49]. These observations are consistent with the EPR spectra of the interfacial film at pH 6 in the present study (Figure 4a). The spectra of the emulsions with CA and RA showed only a slight deviation from the Cu(II) spectrum. Thus, it can be assumed that, at pH 6, the carboxyl groups of CA and RA coordinate the Cu(II) present. For alkaline pH values, Pirker et al. showed that other complexes are formed that predominantly mononuclearily bind the Cu(II). The analyses of rotational correlation times, performed by Pirker et al., also showed an increasing molecular mass at alkaline pH values. This led these to assume that di- or polymeric PCDs complexed the Cu(II) [48]. Due to the deprotonation of the hydroxyl groups under alkaline conditions, PCDs can form Cu(II) complexes on the deprotonated phenolic hydroxyl groups [50]. Therefore, complexes with different chemical structures are formed, depending on the pH value; this results in other binding sites, which can be shown through clear differences in the EPR spectra. As hypothesized, the deprotonation at pH 9 leads to the formation of strong Cu(II)-PCD complexes, while at pH 6, only weaker complexes via the carboxyl group are assumed to be formed.

### 3.3. Iron Reducing Power of β-Lg-PCD Mixtures

**Figure 5:**
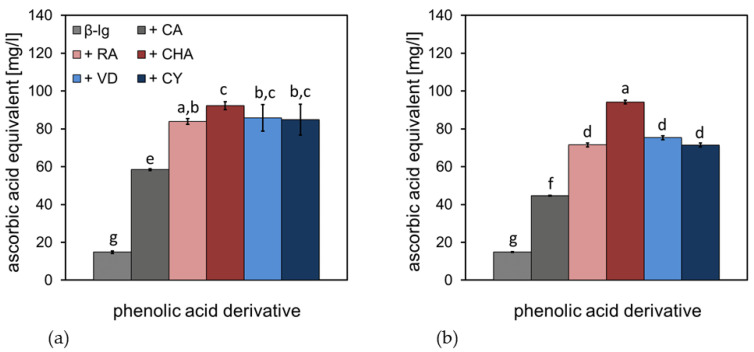
Fe(III)-reducing power calculated as ascorbic acid equivalents (**a**) of β-Lg-PCD mixtures at pH 6 and (**b**) at pH 9; the letters (a–g) are describing the statistical homogeneous groups without significant differences (*p* > 0.05) for (**a**,**b**).

The Fe(III)-reducing power of β-Lg-PCD mixtures was investigated by a photometric measurement at pH 6 and 9; this was performed in order to assess the impact of structural effects and pH on pro-oxidant efficacy by maintaining the redox cycle, due to electron donation. The results, calculated as ascorbic acid equivalents (AAE), are shown in Figure 5. For both pH conditions, the pure β-Lg solution showed a low iron-reducing power with an AAE of approximately 15 mg/L. The addition of CA increased the iron-reducing power to approximately 60 mg/L AAE at pH 6 and 45 mg/L AAE at pH 9. The higher molecular weight of the PCD (RA, CHA, VD and CY) led to a further increase in the value to 80–90 mg/L AAE at pH 6, with CHA showing the highest iron-reduction capacity. At pH 9, the increase in the iron-reducing power, due to PCD addition, was less pronounced than at pH 6. The RA, VD, and CY were approximately 70 mg/L AAE; only CHA increased to 90 mg/L AAE.

At both pH conditions, there was a trend in molecules with a low molecular weight, such as CA, donating fewer electrons; this reduced transition metals, such as iron, rather than molecules with a higher molecular weight, such as RA, CHA, VD, and CY. This is because molecules with a higher molecular weight can have more conjugated π-electrons and are therefore more resonance stabilized, donating more electrons for Fe(III) reduction. Yen et al. (2002) described the relationship between the ability to complex metals and donate electrons, and hence the metal reducing power of ascorbic acid and gallic acid [51]. These authors concluded that metal complexation simultaneously decreased the reduction of transition metals and, therefore, their chemical reactivity, especially in lipid oxidation [34,51,52]. The decrease in iron reducing power is, therefore, consistent with the EPR measurements of the present study (Figure 4), showing a significant metal complexation at pH 9, which was not the case at pH 6. In addition, there is the possibility that PCDs, covalently bound to the protein, or even polymerized PCDs at pH 9, can only donate protons to a limited extent and can, therefore, reduce less Fe(III).

In summary, PCDs formed various complexes with transition metals. It is suspected that at pH 6, partially weak complexes are formed with the carboxyl groups of the PCDs and at pH 9, stronger complexes were formed via the deprotonated hydroxyl groups of the catechol structure of the PCDs. The strong complexation of transition metals at pH 9 contributes to the fact that complexed transition metals no longer take part in the redox cycle, thus inhibiting the reduction of transition metals to pro-oxidant compounds.

### 3.4. Formation of Oxidation Products during Storage

**Figure 6:**
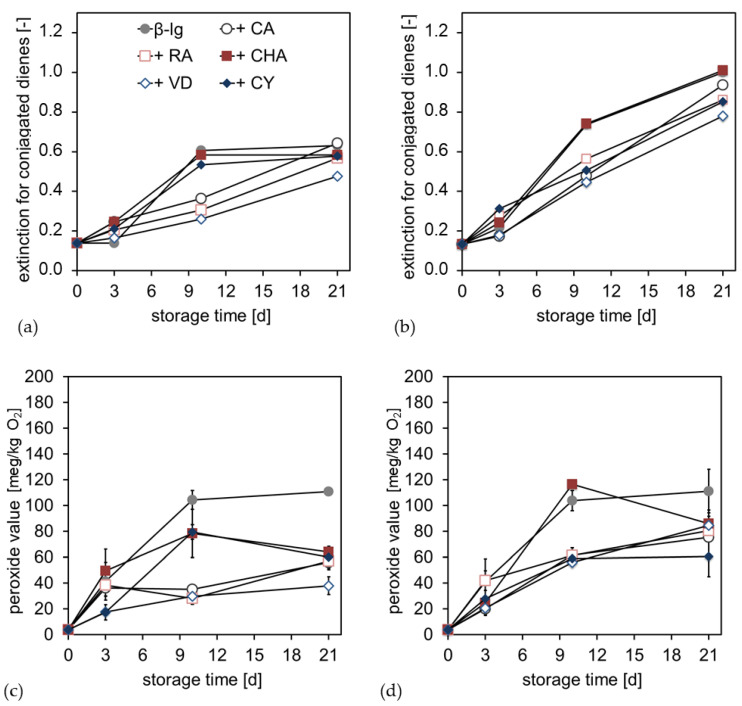
Extinction of the emulsion oil extract at 233 nm as an indicator for the formation of conjugated dienes within the emulsion (**a**) at pH 6 and (**b**) at pH 9; peroxide values for emulsions stabilized by different PCD and stored for 21 days (**c**) at pH 6 and (**d**) at pH 9.

In order to consider the antioxidant and pro-oxidant properties of the PCD–β-Lg interfacial films, a storage experiment was performed with a linseed oil emulsion. In the first step, primary oxidation products during storage were investigated. These were initially conjugated dienes (CD) (Figure 6a,b), which in turn react further to form hydroperoxides (Figure 6c,d). At pH 6 and 9, an increase in conjugated dienes in the emulsion had already occurred within the first three days. At pH 6, the pure β-Lg emulsion and the samples with CHA and CY showed a stronger increase in CD than the samples with CA, RA, and VD. After completion of the storage test, however, the CD contents converge again. Even at pH 9, the CD content increased faster with the pure β-Lg emulsion, as well as with CHA, than with the other PCD; there was a similar final level at the end of the storage time.

With the formation of CD, an increase in the peroxide value (POV) could also be estimated for pH 6 and 9. Considered over the storage time, the POVs were highest for the pure β-Lg emulsion. At pH 6, CHA and CY again showed a faster increase in POV than CA, RA, and VD. However, over the storage time, POV decreased again, so that at the end of the storage time, POV for emulsions with PCDs were between 40 and 60 meq/kg O_2_, while the emulsion without PCDs was approximately 110 meq/kg of O_2_. At pH 9, there was a significant increase in the POV of the emulsion with CHA, at about 120 meq/kg of O_2_ on day 9. The other PCDs were comparatively low on day 9, at 60 meq/kg of O_2_. The final level, at the end of the storage period, was higher at pH 9 than at pH 6, at about 60 to 80 meq/kg of O_2_. The pure β-Lg emulsion was again at approximately 110 meq/kg of O_2_. Thus, for both pH conditions, a stagnation or a decrease in POV could be observed in the second half of the storage time, with the POV of the emulsions with PCDs, generally, significantly lower than without PCDs.

The increase in CD during the storage time showed that the double bonds of polyunsaturated fatty acids were shifted due to oxidation processes, resulting in conjugated dienes. These dienes react further in the course of chain propagation to form hydroperoxides [15,53]. The increase in CD indicated that lipid oxidation progress occurs in all samples, reaching a plateau only for the emulsion at pH 6, without PCD sor with CHA or CY. Within this plateau of CHA and CY, the degradation of hydroperoxides had already occurred. For these compounds, this indicated that secondary oxidation products were increasingly formed [15]. It was notable that CHA and CY were the compounds with the largest π-electron systems and, therefore, showed the greatest resonance stabilization. In addition, at pH 9, the emulsions with CHA and CY indicated a trend in forming a plateau, in addition to a degradation of the hydroperoxides; meanwhile, an increase in hydroperoxide formation was still observed with the other samples.

**Figure 7:**
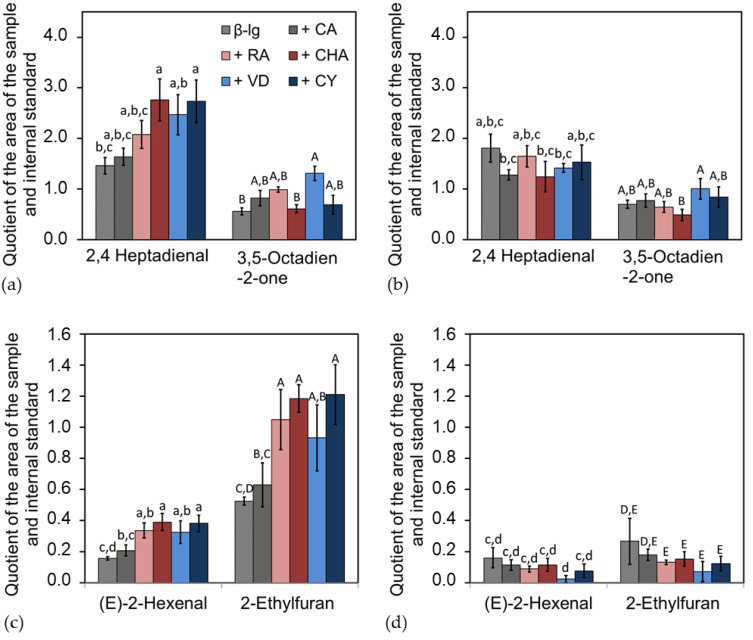
Oxidation products of the linseed oil emulsion after 21 d of storage time, shown as quotient of the internal standard: Quantitative main components of the secondary oxidation products of linolenic acid (**a**) at pH 6 and (**b**) at pH 9; the letters (a–c and A–B) are describing the statistical homogeneous groups without significant differences (*p* >0.05) for (**a**,**b**); and the further reaction of a secondary oxidation product ((E)-2-hexenal) to a tertiary oxidation product (2-ethylfuran) (**c**) at pH 6 and (**d**) at pH 9; the letters (a–d and A-E) are describing the statistical homogeneous groups without significant differences (*p* >0.05) for (**c**,**d**).

At the end of the storage time, the emulsions were analyzed for secondary and tertiary oxidation products. Among other compounds, 2,4-heptadienal and 3,5-octadien-2-one (Figure 7a,b), as well as 2-hexenal and its reaction product ethylfuran (Figure 7c,d), were found to be the main components in terms of concentration.

For all samples, significantly more oxidation products were formed during the storage of emulsions at pH 6 than at pH 9. The emulsions with PCDs at pH 6 showed a significant increase in oxidation products, compared to the emulsion without PCDs. There were shifts in the composition of the oxidation products, e.g., CHA and CY promoted the formation of 2,4-heptadienal, while less 3,5-octadien-2-one was formed. In addition, CA led to a less pronounced increase in oxidation products than the PCD RA, CHA, VD, and CY with high molecular weights. For the emulsions at pH 9, no significant differences were observed in the formation of the oxidation products, with and without PCDs. The most significant difference between the emulsion at pH 9 and pH 6 could be seen in the formation of the 2-hexenal. In emulsions at pH 6 with PCDs, significantly more 2-hexenal was formed compared to pH 9, which, in turn, reacted further to a higher extent of 2-ethylfuran; therefore, approximately five times the amount of ethylfuran was measured at pH 6, compared to pH 9.

The formed primary, secondary, and tertiary oxidation products showed that the pH of the emulsion had the most intense impact on the lipid oxidation. The molecular weight and structural properties of the PCDs equally had an impact on lipid oxidation products.

However, at the end of the storage period, significantly more secondary and tertiary oxidation products were formed in the emulsions at pH 6 with PCDs than when without the addition of PCDs. For the emulsion at pH 9, no significant differences in the formation of secondary and tertiary oxidation products could be determined, in comparison to with or without PCD. Thus, the reaction conditions at pH 6 induced a pro-oxidant effect on the emulsion. This pro-oxidant effect may be due to the following causes: As is obvious from Figure 4 and Figure 5, PCDs have a high potential at pH 6 to reduce Fe(III), but a low ability to complex transition metals. Reduced transition metals, such as Fe(II), are known to react intensively with hydroperoxides, leading to radical formation during autoxidation. Thus, for the iron-containing linseed oil emulsion, it is suspected that the hydroperoxides, formed in the emulsion at pH 6, decompose into secondary oxidation products faster than the hydroperoxides formed in the emulsions at pH 9 do. Therefore, the POV of the emulsion at pH 6 is lower than at pH 9.

For pH 9, it was shown in Figure 5 that PCDs had a significantly low ability to reduce Fe(III). In addition, Figure 4 showed the high ability of PCDs to complex transition metals. The experiments described by Iwahashi et al. indicated that Fe(III) forms a complex with caffeic acid, which cannot be reduced under the present conditions (pH 7.4 in this study) [54]. The redox cycle of the transition metals would thus be interrupted, and the further reaction of the hydroperoxides to secondary oxidation products would not be promoted.

The high content of tertiary oxidation products in the emulsions at pH 6 with PCDs indicates a progressive lipid oxidation, compared to the emulsion at pH 9. According to Grebenteuch et al., the formation of tertiary oxidation products is an indicator for a more advanced lipid oxidation; this should be considered to be differentiated to secondary oxidation products in the context of the evaluation [18].

Hence, the use of PCD seems to promote not only the formation of secondary oxidation products, but also their further reaction to tertiary oxidation products. 2-Ethylfuran, as a tertiary oxidation product, can be formed from a secondary oxidation product, such as the unsaturated aldehyde 2-hexenal. Adams et al. showed, for these compounds, that this reaction can be further triggered in the presence of amino acids, peptides, and proteins [55]. Thus, it is likely that the interfacial protein also has a catalytic effect on the formation of 2-ethylfuran. Effectively, it is not possible to differentiate whether the increased 2-ethylfuran formation is also promoted by PCDs or whether the increased formation of secondary oxidation products at pH 6, in the presence of the interfacial protein film, is the cause of the 2-ethylfuran formation.

In summary, the addition of PCDs to the emulsions at pH 6 had a pro-oxidant effect. The further reaction of primary oxidation products to secondary and possibly tertiary oxidation products is promoted by the presence of PCDs in the emulsion at pH 6. In contrast, there are no significant differences at pH 9. The pro-oxidant behavior of the PCDs could be attributed to their ability to reduce transition metals, making them pro-oxidantly effective. At pH 9, this observation occurs hardly at all, as transition metals can be complexed via the hydroxyl groups of the PCDs and lose their pro-oxidant effect when being complexed, as they can no longer be reduced.

## 4. Conclusions

In this study, the pH and polarity of PCDs were found to have a significant effect on their antioxidant/ pro-oxidant activity at the interfacial protein film.

As hypothesized, the polarity and, therefore, the partitioning behavior of the PCDs, determined whether radicals present could be scavenged from the continuous aqueous phase or the disperse lipid phase of the emulsion. More hydrophobic PCDs could only penetrate the interfacial protein film and scavenge radicals in the lipid phase when they were not electrostatically repelled from it. Polymerized PCDs, as is likely at pH 9, are sterically hindered from penetrating the interfacial protein film and, therefore, do not enter the oil phase. Monomers present in protonated PCDs at pH 6 showed the ability to reduce transition metals to pro-oxidant-active oxidation states via their capability to split off electrons, as hypothesized. They also showed a low complexation ability for transition metals, which likely occurs via the carboxyl groups of the PCDs. Consequently, PCDs have a pro-oxidant effect in an emulsion. Polymeric deprotonated PCDs at pH 9 reduced the transition metals present to a lesser extent than at pH 6, as hypothesized. At pH 9, the hydroxyl groups of the catechol structures of the PCDs were partially deprotonated and formed stable complexes with the transition metals over them. The transition metals could, then, no longer be reduced and, accordingly, they could no longer have a pro-oxidant effect on the lipid phase.

The results suggest that the technological use of PCDs as an antioxidant should definitely be performed, considering the food prevailing conditions. For example, a pro-oxidant effect of PCDs can be expected at low pH values and in the presence of transition metals. Alternatively, provided that PCDs occur naturally in food, it is conceivable to selectively induce covalent bonds and polymerization reactions to control their pro-oxidant properties.

In the follow-up research, it would be interesting to look at how the system behaves if, instead of varying the pH, oxidative conditions can be created by other means. The use of enzymes or electrochemical potentials would be conceivable here. Thus, the impact of electrostatic effects, due to the different protonation at pH 6 and 9, could be minimized, and the results could be related more concretely to the predominant bond types.

## Data Availability

The data are contained within this article.
